# The Effect of Serum 25-Hydroxyvitamin D Concentrations on Elevated Serum C-Reactive Protein Concentrations in Normal Weight, Overweight and Obese Participants of a Preventive Health Program

**DOI:** 10.3390/nu8110696

**Published:** 2016-11-04

**Authors:** Silmara S. B. S. Mastroeni, Lalani L. Munasinghe, Truong-Minh Pham, Sarah A. Loehr, John Paul Ekwaru, Marco F. Mastroeni, Paul J. Veugelers

**Affiliations:** 1 Department of Physical Education, University of Joinville Region, Rua Paulo Malschitzki, No. 10, Joinville 89.219-710, Brazil; silmara.mastroeni@univille.br; 2 Population Health Intervention Research Unit, School of Public Health, University of Alberta, 3-50 University Terrace, 8303-112 St., Edmonton, AB T6G 2T4, Canada; lalani.m@ualberta.ca (L.L.M.); truongminh.pham@ualberta.ca (T.-M.P.); Sarah.Loehr@ualberta.ca (S.A.L.); ekwaru@ualberta.ca (J.P.E.), marco.mastroeni@univille.br (M.F.M.); 3 Post-graduation Program in Health and Environment, University of Joinville Region, Rua Paulo Malschitzki, No. 10, Joinville 89.219-710, Brazil

**Keywords:** serum 25-hydroxyvitamin D, vitamin D, C-reactive protein, inflammation, obesity, cardiovascular disease

## Abstract

The hypothesized effect of vitamin D on C-reactive protein (CRP) has received substantial attention as a potential means to alleviate the risk for cardiovascular disease. However, observational studies have been inconsistent in their reporting of associations between serum 25-hydroxyvitamin D (25(OH)D) and CRP concentrations, and trials and meta analyses have been inconsistent in their conclusions regarding the effect of vitamin D supplementation on CRP concentrations. These supplementation trials were mostly conducted among patients with more or less inflammatory complications and did not consider potential distinctive effects by weight status. To further our understanding of the potential influences of vitamin D on CRP, we analyzed longitudinal observations of 6755 participants of a preventative health program. On average, serum 25(OH)D concentrations increased from 88.3 to 121.0 nmol/L and those of CRP decreased from 1.7 to 1.6 mg/L between baseline and follow up. Relative to obese participants without temporal increases in 25(OH)D, those who showed improvements of <25, 25–50, 50–75, and more than 75 nmol/L at follow up were 0.57 (95% confidence interval: 0.37–0.88), 0.54 (0.34–0.85), 0.49 (0.30–0.80), and 0.48 (0.29–0.78) times as likely to have elevated CRP concentrations (≥1 mg/L), respectively. These associations were less pronounced and not statistically significant for normal weight and overweight participants. Herewith, the findings suggest that promotion of adequate serum 25(OH)D concentrations among obese individuals along with healthy lifestyles may alleviate the public health burden associated with cardiovascular disease.

## 1. Introduction

C-reactive protein (CRP) is a novel risk factor for cardiovascular disease (CVD) [1,2,3]. It is a positive acute phase protein produced by hepatocytes in low quantities under normal conditions and in higher quantities in response to acute phase stimuli such as inflammation, infections, allergies and trauma [1,3,4]. As a chronic low-grade inflammatory state and an established risk factor for CVD, excess body weight increases serum CRP concentrations [2,5,6,7]. Reducing serum CRP concentrations has therefore been considered as an approach to decrease the risk for CVD [8,9], particularly among obese individuals.

Vitamin D is synthesized in the skin when exposed to sunlight, and can be ingested through the diet and supplementation [10]. Where the role of vitamin D in sustaining calcium homeostasis and bone health is well established [10,11], vitamin D has also been suggested to have other health benefits such as the prevention of CVD [2,3,8,12]. CRP may be involved in one or more of the various pathways through which vitamin D moderates the risk for CVD. The potential importance of vitamin D for serum CRP concentrations has received great attention in recent years. Observational studies have reported, though not consistently, associations between serum 25-hydroxyvitamin D (25(OH)D) and CRP concentrations [13,14,15]. Randomized controlled trials (RCT) [16,17,18,19,20,21,22] have produced inconsistent findings with respect to the effect of vitamin D supplementation regimens on serum CRP concentrations. A meta-analysis focusing on supplementation trials among overweight and obese individuals concluded the absence of a significant influence of vitamin D supplementation on serum CRP concentrations [23]. A meta-analysis of supplementation trials published since 2010 concluded the presence of such an influence [24], suggesting the influence of vitamin D on CRP may differ by body weight. In addition, because the participants of most of the RCTs were patients with more or less inflammatory complications, their results are difficult to generalize to the general population [24], prompting the need for supplementation studies in community samples. Furthermore, because RCTs quantify the influence of vitamin D supplementation regimens on changes in serum CRP concentrations, they do not provide a good understanding of how temporal changes in serum 25(OH)D concentrations affect the risk for reaching adverse CRP concentrations. The latter risk estimates may be more informative for clinical and public health decision making as they also consider the influence of sun exposure and diet. Therefore, the objective of this study was to examine whether temporal increases in serum 25(OH)D concentrations are paralleled by a reduction in the risk for reaching adverse CRP concentrations in a community sample. This objective was also addressed separately for normal weight, overweight and obese subjects.

## 2. Methods

### 2.1. Study Design and Participants

The data for the present study were obtained from the Pure North S’Energy Foundation (PN), a not-for-profit organization, in Calgary, Alberta, Canada that offers a preventative health program to community volunteers. Details of the program have been previous publish elsewhere [25,26] and further information can be found on the PN website (http://purenorth.ca/pure-north-program/). In brief, established in 2007, this program employs health professional to offer lifestyle counseling to volunteer participants. At enrollment, all participants complete a demographic and lifestyle questionnaire, and provide information regarding their medical history and medication and supplement use. Blood pressure and anthropometric measurements are taken by trained professionals, and blood is drawn for the assessment of several biomarkers including serum 25(OH)D and high sensitive CRP (hs-CRP). The information collected is used to guide lifestyle counseling. Vitamin D supplementation is encouraged given Canada’s Northern latitude, limited sunlight and inadequate cutaneous synthesis of vitamin D. Follow-up visits, which include both assessments and counseling, are scheduled annually. Data collected from October 2007 through April 2014 were used for the current analyses. Participants signed and granted written informed consent for the use of their relevant information for research purposes. All data were anonymized by PN prior to it being transferred to the University of Alberta for data analyses. Ethical approval was obtained from the Human Research Ethics Board at the University of Alberta (Pro00028578).

**Figure 1:**
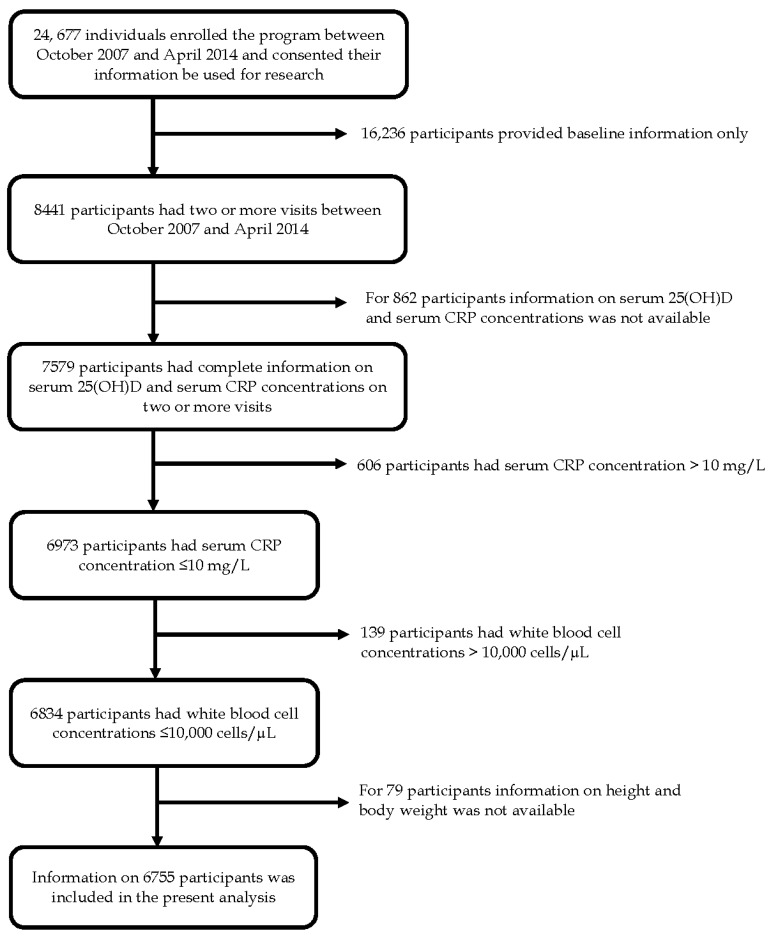
Flow chart of adult participants of the Pure North preventive health program.

Figure 1 provides a flow chart of the participants in the PN program included in the present analysis. A total of 7579 adult participants had at least two measures of serum 25(OH)D concentrations and serum CRP concentrations, one at baseline and one or more follow-up visits. The focus of the present study is chronic inflammation and CVD risk; therefore, observations were excluded from the analyses if serum CRP concentrations were in excess of 10 mg/L (*n* = 606) due to the possibility that such a high concentration was caused by acute infections or inflammations rather than chronic inflammation [27], or if white blood cell concentrations were in excess of 10,000 cells/µL (*n* = 139) due to the possibility of an acute infection. Observations were also excluded if information on weight or height was missing (*n* = 79). The final analysis included data from 6755 participants contributing to 6755 baseline visits and 10,383 follow-up visits (Figure 1).

### 2.2. Serum CRP Concentrations

High-sensitive CRP, which is sensitive to the detection of small changes at low serum CRP concentrations (below 10 mg/L) [28], was determined using an immunoturbidimetric principle on an automated analyzer with the inter-assay CV of 2.5%. Serum CRP concentrations were categorized into three groups as “low CVD-risk (<1 mg/L)”, “average CVD-risk (1 to <3 mg/L)” and “high CVD-risk (≥3 mg/L)” as per established criteria [27] though alternative categorizations have also been published [29]. 

### 2.3. Serum 25(OH)D Concentrations

Serum 25(OH)D concentrations were determined using DiaSorin^®^ Liason chemiluminescent immunoassay with an inter-assay coefficient of variation (CV) of 11%. Baseline 25(OH)D were categorized into five groups (<50, 50 to <75, 75 to <100, 100 to <125 and ≥125 nmol/L) based on their distribution against serum CRP. The change in 25(OH)D was calculated by subtracting the baseline concentration from the follow-up concentration. Changes were categorized into five groups (no improvement, increased by <25, increased by 25 to <50, increased by 50 to <75 and increased by ≥75 nmol/L).

### 2.4. Body Weight Status 

To determine body weight status, body mass index (BMI) was calculated for each participant as weight in kg/height^2^ (kg/m^2^); BMI was then categorized as “underweight (<18.5 kg/m^2^)”, “normal weight (18.5 to <25 kg/m^2^)”, “overweight (25 to <30 kg/m^2^)”, and “obesity (≥30 kg/m^2^)” [30]. Due to low prevalence of underweight participants, underweight and normal weight individuals were combined into one group in the regression analysis.

### 2.5. Potential Confounding Variables

Low-density lipoprotein (LDL) cholesterol, age, gender, blood pressure, smoking, alcohol consumption, and physical activity have all been associated with CVD and were considered as potential confounding variables in the present study. Automated Cobas^®^ 8000 Modular Analyzer Series was used to measure concentrations of serum triglycerides (inter-assay CV = 2%), total cholesterol (inter-assay CV = 1.5%), and high-density lipoprotein (HDL; inter-assay CV = 2%). LDL concentrations were calculated using the formula, “LDL = Total Cholesterol − HDL − (Triglycerides/2.2)”, and categorized as “Normal (<2.6 mmol/L)” and “Elevated (≥2.6 mmol/L)”. Blood pressure status was defined as “Normal” if systolic and diastolic pressures were <140/90 mmHg, and as “Elevated” if they were ≥140/90 mmHg or if the participant was using an anti-hypertensive medication. Information on age, gender, smoking status (“never smoker”, “past smoker”, and “current smoker”), alcohol consumption and physical activities for a typical week (light, moderate and vigorous activities) was ascertained from the self-reported lifestyle questionnaire. The alcohol consumption status was defined as “non-drinker” for those who reported drinking <2 glasses per week and “drinker” for those who drank ≥2 glasses per week. Physical activity levels were determined by estimating the metabolic equivalent of task (MET) per week for each physical activity recorded by participants on their questionnaires. MET values were then multiplied by the time participants spent performing those activities per week (MET * hours per week), and the total for each week was categorized as “low (<10 MET hours/week)”, “moderate (10 to <20 MET hours/week)” and “high (≥20 MET hours/week)”. MET hours per week at baseline were subtracted from the MET hours per week at the follow-up visit to determine change in physical activity. Change in physical activity was categorized as “Negative change” if MET hours per week were greater at baseline than follow-up, “No change” if MET hours per week remained constant and “Positive change” MET hours per week were greater at follow-up than baseline.

### 2.6. Statistical Analyses

Descriptive statistics were presented for both baseline visits and the last follow-up visits. Serum CRP concentrations of <1 mg/L representing “low CVD risk” were contrasted with serum CRP concentration of ≥1 mg/L representing “elevated CRP” that combines the categories “average CVD-risk” and “high CVD-risk” associated with elevated CRP [4]. Multiple logistic regression analyses with mixed effects to accommodate the repeated measures of participants were used to quantify the associations of 25(OH)D at baseline and changes in 25(OH)D during follow-up with elevated CRP at follow-up. These regression analyses were adjusted for elevated CRP at baseline, baseline LDL-cholesterol, age, gender, baseline blood pressure, baseline smoking status, baseline alcohol consumption status, baseline physical activity level and change in physical activity level. These associations were quantified separately for normal weight, overweight and obese individuals. Missing observations for confounding variables were treated as separate covariate categories and presented as “Missing”. All statistical analyses were performed using Stata, version 14.0 (Stata Corp, College Station, TX, USA) with statistical significance at 0.05.

## 3. Results

Participant characteristics at baseline and at the last follow-up visit are presented in Table 1. Mean serum 25(OH)D concentrations increased from 88.3 nmol/L at baseline to 121.0 nmol/L at the last follow-up visit, with a concurrent decrease in mean CRP from 1.7 mg/L at baseline to 1.6 mg/L at the last follow-up visit. The prevalence of elevated CRP concentrations (serum CRP concentrations of 1 mg/L or more) decreased from 53.0% to 48.8%. The median time between baseline and the last follow up visit was 1.1 year.

Table 2 depicts the results of the longitudinal analysis of the risk for elevated CRP concentrations at follow-up. Baseline measures including lower 25(OH)D, elevated CRP, elevated LDL-cholesterol, older age, female gender, being overweight or obese, and being a current smoker were associated with an increased risk for elevated CRP at follow-up. Baseline physical activity levels and increases in physical activity were inversely associated with the risk for elevated CRP.

Table 3 shows the associations of baseline 25(OH)D concentrations and temporal changes in 25(OH)D concentrations with the risk for elevated CRP concentrations at follow-up by body weight status adjusted for the same confounders as considered in the above analyses and listed in Table 2. For underweight and normal weight participants, neither baseline 25(OH)D concentrations nor changes in 25(OH)D concentrations were associated with the risk for elevated CRP at follow-up. Overweight participants with baseline 25(OH)D concentrations above 75 nmol/L had a lower risk of elevated CRP at follow-up relative to those with lower baseline 25(OH)D concentrations. For obese participants, both baseline 25(OH)D concentrations and changes in 25(OH)D concentrations were inversely associated with the risk for elevated CRP concentrations at follow-up (Table 3).

In the above analyses, we considered serum CRP concentration of ≥1 mg/L as “elevated CRP” to capture both average CVD-risk (≥1 mg/L and <3 mg/L) and high CVD-risk (≥3 mg/L). A comparison considering serum CRP concentration of ≥3 mg/L as “elevated CRP” and <3 mg/L as “not elevated” revealed similar findings in that the associations of baseline 25(OH)D concentrations and temporal changes in 25(OH)D concentrations with the risk for elevated CRP concentrations at follow-up were statistically significant for obese participants, but not for underweight and normal weight participants. These results are included in Supplementary Table S1.

We considered various confounders in the analyses presented in Table 2 and Table 3. Vitamin D supplement use and dose were not considered, as they do not qualify as confounders. A repeat of the analyses presented in Table 2 and Table 3 with further consideration of supplement use and dose revealed very similar estimates for the associations of baseline 25(OH)D concentrations and temporal changes in 25(OH)D concentrations with the risk for elevated CRP concentrations at follow-up.

## 4. Discussion

We revealed that higher serum 25(OH)D concentrations at baseline were associated with a reduced risk for elevated CRP concentrations at follow-up among overweight and obese participants, and that temporal increases in serum 25(OH)D concentrations were paralleled with a reduction in the risk for elevated CRP concentrations among obese participants. No statistically significant associations of 25(OH)D with risk for elevated CRP were observed for underweight and normal weight participants. To the best of our knowledge, this is the first study that shows that changes in the risk for elevated CRP in response to temporal changes in 25(OH)D concentration differ by body weight.

Several observational studies, though not consistently, reported inverse associations of serum 25(OH)D concentrations with CRP concentrations [13,14,15,31,32]. Amer and Qayyum [14] observed the inverse association between 25(OH)D and CRP concentrations to exist for individuals with serum 25(OH)D concentrations ≤52.5 nmol/L but not for those with higher 25(OH)D concentrations. As individuals with excess body weight, on average, have lower serum 25(OH)D concentrations [33], one may speculate that the subgroup with serum 25(OH)D concentrations ≤52.5 nmol/L in the analyses by Amer and Qayyum [14] included relatively more individuals with excess body weight. One may further speculate that the revelation of the present study that the association between 25(OH)D and CRP exists for obese subjects and not for normal weight individuals, explains why the inverse association was shown for the subgroup with serum 25(OH)D concentrations ≤52.5 nmol/L and not for the subgroup with 25(OH)D >52.5 nmol/L. In addition, Bellia et al. [13] had shown this inverse association of 25(OH)D with CRP to exist for obese subjects in a cross sectional study. The conclusion arising from the meta-analysis of vitamin D supplementation trials among overweight and obese individuals by Jamka et al. [23] seems to contradict the findings of these observational studies and of the present study that temporal increases in 25(OH)D concentrations are paralleled by decreases in CRP among obese subjects. Jamka et al. [23] had observed a reduction in CRP associated with supplementation but this was not statistically significant. One may speculate that this is attributable to sample size and statistical power as the analysis by Jamka et al. [23] were based on observations of 1955 subjects from 13 RCTs, whereas the present analyses included observations of 6755 participants. However, various other differences between the studies exist that may explain the differences in findings. The most prominent difference is that we analyzed changes in 25(OH)D concentrations which is an objective measure not affected by compliance to supplementation regimens, and includes the contribution of vitamin D from sunlight and diet. The conclusions arising from the meta analyses by Chen et al. [24] seems consistent with those of the present study, despite the fact that RCTs considered in Chen et al. [24] mostly consisted of patients with inflammatory complications and our analyses were based on a community sample of volunteers participating in a preventive health program. The present study herewith illustrates that the conclusions arising from RCTs in selected patients groups are also applicable to the population at large and those with obesity in particular.

Heaney et al. [34] showed that associations of 25(OH)D concentrations with insulin resistance and blood pressure exists but only for the range of approximately 40 to 90 nmol/L of 25(OH)D. No associations were found below and above these response thresholds. The response thresholds for the association of 25(OH)D with inflammation and CRP concentrations have yet to be investigated and reported. These thresholds are likely distinct from those for insulin resistance and blood pressure, given the conclusion of a recent review of the literature that vitamin D effects different health conditions at distinct serum 25(OH)D concentrations [35]. In the absence of knowledge of these thresholds, one should be cautious in the application of linear regression which assumes a linear relationship between the exposure (25(OH)D) and the outcome (CRP) to the full range of the exposure (25(OH)D). This concern was mitigated in the present study by examining the risk for elevated CRP concentrations and employing established thresholds for CRP [27]. These thresholds allowed us to contrast CRP concentrations associated with low CVD-risk (<1 mg/L) with CRP concentrations associated with elevated CVD risk (average CVD-risk (1 to <3 mg/L) and high CVD-risk (≥3 mg/L) combined) [27]. As such, the estimates for risk for elevated CRP may also be interpreted as risk for CVD [27]. It has been previously suggested that the responsible mechanism may involve the inhibitory effects of vitamin D on synthesizing the primary stimulant of CRP production in the liver [36,37] or the anti-inflammatory effect of vitamin D [38].

In addition to excess body weight, the present study confirmed that many of the conventional CVD risk factors including elevated LDL-cholesterol [1], increasing age [2], smoking [1,2], low level of physical activity [39,40] and overweight/obesity [2] were associated with an increased risk for elevated CRP. However, no statistically significant associations of blood pressure and alcohol consumption were observed. In addition, where male gender is an established risk factor for CVD [1], we observed a higher risk for elevated CRP concentrations among females.

The longitudinal design, large sample size and the wide range of serum 25(OH)D concentrations are strengths of the present study. As limitations we acknowledge that this study was conducted among volunteer participants of a preventive health program. These participants may not be representative of the general population. The preventive health program not only encourages supplementation with vitamin D, but rather healthy lifestyles in general. In this regard, we did report that physical active levels improved during follow-up. However, other lifestyle changes may also have occurred, though not recorded, and thus not allowing us to adjust for this in the analyses. Other limitations relate to inaccuracies of the vitamin D assay and the fact that vitamin D was routinely collected and subjected to the assay rather than batch-wise. The absence of information on serum parathyroid hormone concentrations as well as missing values for the confounding variables may have contributed to a partial adjustment for confounding. These limitations underline that caution is warranted in the interpretation and generalization of the present findings.

## 5. Conclusions

In conclusion, we revealed high serum 25(OH)D concentrations at baseline and prospective improvement in 25(OH)D concentrations decrease the risk for elevated serum CRP concentration in individuals with excess body weight but not in those with normal weight. In Canada, where 20% of adults are obese [41] and 39% are vitamin D insufficient [42], adverse CRP concentrations are common, and CVD constitutes an enormous public health burden, promotion of adequate serum 25(OH)D concentrations along with healthy lifestyles that include healthy diets, physical activity, and abstaining from smoking may alleviate the public health burden associated with CVD.

## Figures and Tables

**Table 1:** Baseline and follow-up characteristics of 6755 study participants.

Characteristic	Baseline	Last Follow-up Visit
**Serum 25(OH)D, nmol/L**		
Mean (SD)	88.3 (42.2)	121.0 (46.2)
Median (IQR)	81.6 (60.5–108.0)	115.0 (88.60–147.0)
**Serum C-reactive protein, mg/L**		
Mean (SD)	1.7 (1.8)	1.6 (1.8)
Median (IQR)	1.0 (0.5–2.2)	0.9 (0.4–2.1)
**Elevated C-reactive protein, %**		
Low-risk (<1 mg/L)	47.0	51.2
Average risk (1–2.99 mg/L)	35.6	32.6
High risk (≥3 mg/L)	17.4	16.2
**Gender (%)**		
Female	52.0	52.0
Male	48.0	48.0
**Age**		
Mean (SD)	50.9 (15.2)	52.4 (15.0)
**Body weight status, %**		
Under weight	1.0	1.0
Normal weight	34.2	34.3
Over weight	38.2	37.7
Obesity	26.6	27.0
Blood pressure, % *		
Normal (<140/90 mmHg)	58.9	53.2
Elevated (≥140/90 mmHg or anti-hypertensive medication use)	36.2	33.6
Missing	4.9	13.2
**Serum LDL-cholesterol, % ^†^**		
Normal (<2.6 mmol/L)	34.3	30.0
Elevated (≥2.6 mmol/L)	61.7	67.3
Missing	4.0	2.7
**Smoking status, %**		
Never smoker	41.5	30.2
Ex-smoker	21.6	15.6
Current smoker	8.7	6.6
Missing	28.2	47.6
**Alcohol consumption status, %**		
Non-drinker	25.3	26.6
Drinker	42.1	41.6
Missing	32.6	31.8
**Physical activity level, %**		
Low	28.3	28.3
Moderate	21.5	21.5
High	21.0	21.0
Missing	29.2	29.2
**Use of vitamin D-containing supplements, %**		
Yes	47.4	74.2
No	35.7	9.6
Missing	16.9	16.2
**Vitamin D dose of the supplements, Median (IQR) IU/day**	3000 (2000–5000)	6000 (4000–9000)

Abbreviations: 25(OH)D, 25-hydroxyvitamin D; LDL-cholesterol, low-density lipoprotein cholesterol; SD, standard deviation; IQR, interquartile range; * Blood pressure status was defined based on blood pressure ≥140/90 mm Hg, or a self-report of taking antihypertensive medications as elevated; ^†^ Elevated LDL-cholesterol was defined as LDL-cholesterol concentration ≥2.6 mmol/L.

**Table 2:** Risk for elevated C-reactive protein concentration (≥1 mg/L) at follow-up among 6755 participants with a total of 10,383 follow up visits.

	# Visits	Univariable model ^§^	*p*	Multivariable model ^§^	*p*
		OR (95% CI)		OR (95% CI)	
**Serum 25(OHD) at baseline, nmol/L**					
<50	1684	ref		ref	
50–<75	2878	0.56 (0.41, 0.76)	**<0.01**	0.72 (0.56, 0.92)	**0.01**
75–<100	2655	0.28 (0.20, 0.38)	**<0.01**	0.61 (0.47, 0.80)	**<0.01**
100–<125	1665	0.24 (0.17, 0.34)	**<0.01**	0.61 (0.45, 0.82)	**<0.01**
≥125	1501	0.18 (0.12, 0.26)	**<0.01**	0.58 (0.42, 0.80)	**<0.01**
**Change in serum 25(OH)D, nmol/L**					
No improvement	2032	ref		ref	
Increase of <25	2370	1.38 (1.09, 1.76)	**0.01**	0.92 (0.74, 1.15)	0.46
Increase of 25–<50	2415	1.23 (0.97, 1.57)	0.09	0.84 (0.77, 1.05)	0.13
Increase of 50–<75	1670	1.37 (1.05, 1.78)	**0.02**	0.88 (0.68, 1.13)	0.31
Increase of ≥75	1896	1.28 (0.99, 1.67)	0.06	0.88 (0.69, 1.13)	0.32
**Serum CRP ≥1 mg/L at baseline**					
No	4965	ref		ref	
Yes	5418	48.29 (37.23, 62.63)	**<0.01**	27.80 (21.95, 35.15)	**<0.01**
Serum LDL-cholesterol at baseline *					
Normal	3441	ref		ref	
Elevated	6511	2.31 (1.87, 2.85)	**<0.01**	1.29 (1.09, 1.53)	**<0.01**
Missing	431	2.97 (1.79, 4.91)	**<0.01**	1.38 (0.92, 2.06)	0.12
**Age at baseline (per 10 years)**	10,383	1.34 (1.25, 1.43)	**<0.01**	1.13 (1.06, 1.19)	**<0.01**
**Gender**					
Female	5188	ref		ref	
Male	5195	1.05 (0.87, 1.28)	0.58	0.75 (0.63, 0.89)	**<0.01**
**Body weight status at baseline**					
Underweight/normal weight	3526	ref		ref	
Overweight	4028	4.88 (3.88, 6.14)	**<0.01**	2.17 (1.79, 2.63)	**<0.01**
Obesity	2829	39.03 (28.35, 53.71)	**<0.01**	5.30 (4.17, 6.74)	**<0.01**
**Blood pressure status at baseline ^†^**					
Normal	5993	ref		ref	
Elevated	3752	3.83 (3.09, 4.76)	**<0.01**	1.15 (0.97, 1.37)	0.12
Missing	638	1.59 (1.03, 2.46)	**0.03**	1.10 (0.78, 1.55)	0.60
**Smoking status at baseline**					
Never smoker	3870	ref		ref	
Past smoker	1996	1.59 (1.22, 2.05)	**<0.01**	1.04 (0.84, 1.29)	0.70
Current smoker	743	1.87 (1.29, 2.70)	**<0.01**	1.70 (1.25, 1.31)	**<0.01**
Missing	3774	1.65 (1.31, 2.08)	**<0.01**	0.91 (0.62, 1.32)	0.62
**Alcohol consumption status at baseline**					
Non-drinker	2273	ref		ref	
Drinker	3966	0.66 (0.52, 0.84)	**<0.01**	0.99 (0.81, 1.21)	0.91
Missing	4144	1.28 (1.00, 1.65)	**0.05**	1.22 (0.96, 1.54)	0.10
**Physical activity level at baseline**					
Low	2637	ref		ref	
Moderate	1975	0.46 (0.35, 0.61)	**<0.01**	0.83 (0.66, 1.04)	0.10
High	1927	0.26 (0.20, 0.35)	**<0.01**	0.75 (0.58, 0.95)	**0.02**
Missing	3844	0.78 (0.61, 1.00)	**0.05**	1.31 (0.87, 1.98)	0.19
**Change in physical activity level**					
Negative change	1984	ref		ref	
No change	394	0.71 (0.46, 1.09)	0.12	0.60 (0.40, 0.89)	**0.01**
Positive change	2464	0.75 (0.59, 0.96)	**0.02**	0.67 (0.54, 0.83)	**<0.01**
Missing	5541	0.98 (0.79, 1.23)	0.87	0.81 (0.64, 1.03)	0.08

Abbreviations: OR, Odds Ratio; 95% CI, 95% Confidence Interval; 25(OH)D, 25-hydroxyvitamin D; CVD, cardiovascular disease; CRP, C-reactive protein; LDL-cholesterol, low-density lipoprotein cholesterol; SD, standard deviation; IQR, interquartile range; ^§^ Adjusted for all covariates in the table; * Elevated LDL-cholesterol was defined as LDL-cholesterol concentration ≥2.6 mmol/L; ^†^ Blood pressure status was defined based on blood pressure ≥140/90 mm Hg, or a self-report of taking antihypertensive medications as “elevated”.

**Table 3:** Risk for elevated C-reactive protein concentration (≥1 mg/L) at follow-up by body weight status.

	Underweight/Normal Weight ^§^ # Follow up Visits = 3526		Overweight and Not Obese ^§^ # Follow up Visits = 4028		Obese ^§^ # Follow up Visits = 2829	
	OR (95% CI)	*p*	OR (95% CI)	*p*	OR (95% CI)	*p*
**Serum 25(OHD) at baseline, nmol/L**						
<50	ref		ref		ref	
50–<75	0.71 (0.42, 1.19)	0.19	0.82 (0.57, 1.20)	0.31	0.59 (0.38, 0.92)	**0.02**
75–<100	0.63 (0.38, 1.05)	0.08	0.58 (0.39, 0.86)	**0.01**	0.66 (0.40, 1.10)	0.11
100–<125	0.67 (0.39, 1.16)	0.15	0.62 (0.39, 0.96)	**0.03**	0.51 (0.27, 0.96)	**0.04**
≥125	0.64 (0.36, 1.14)	0.13	0.69 (0.43, 1.13)	0.14	0.35 (0.17, 0.73)	**<0.01**
**Change in serum 25(OH)D, nmol/L**						
No improvement	ref		ref		ref	
Increase of <25	1.16 (0.79, 1.68)	0.45	0.90 (0.64, 1.27)	0.55	0.62 (0.39, 1.00)	**0.05**
Increase of 25–<50	1.12 (0.76, 1.67)	0.56	0.79 (0.55, 1.12)	0.18	0.58 (0.35, 0.96)	**0.03**
Increase of 50–<75	1.25 (0.82, 1.91)	0.30	0.86 (0.58, 1.26)	0.43	0.54 (0.32, 0.92)	**0.02**
Increase of ≥75	1.40 (0.92, 2.13)	0.12	0.82 (0.56, 1.20)	0.30	0.52 (0.31, 0.89)	**0.02**

Abbreviations: OR, Odds Ratio; 95% CI, 95% Confidence Interval; CVD, cardiovascular disease; CRP, C-reactive protein; LDL-cholesterol, low density lipoprotein cholesterol; ^§^ Adjusted for elevated CRP at baseline, LDL-cholesterol at baseline, age at baseline, gender, blood pressure status baseline, smoking status at baseline, alcohol consumption status at baseline, and physical activity level at baseline, and change in physical activity level in addition to variables in the table.

## Supplementary Materials

The following are available online at http://www.mdpi.com/2072-6643/8/11/696/s1, Table S1: Risk for elevated C-reactive protein concentration (≥3 mg/L) at follow-up by body weight status at baseline.
