# Supplementary material for: The Effect of Serum 25-Hydroxyvitamin D Concentrations on Elevated Serum C-Reactive Protein Concentrations in Normal Weight, Overweight and Obese Participants of a Preventive Health Program
Source: Nutrients. 2016 Nov 4;8(11):696. doi: 10.3390/nu8110696 (PMC5133083; doi:10.3390/nu8110696)
Supplement: Supplementary file 1 [file nutrients-08-00696-s001.docx]

Supplementary Materials: The Effect of Serum 25-Hydroxyvitamin D Concentrations on Elevated Serum C-Reactive Protein Concentrations in Normal Weight, Overweight and Obese Participants of a Preventive Health Program

Silmara S. B. S. Mastroeni, Lalani L. Munasinghe, Truong-Minh Pham, Sarah A. Loehr, John Paul Ekwaru, Marco F. Mastroeni and Paul J. Veugelers

**Table S1.** Risk for elevated C-reactive protein concentration (≥3 mg/L) at follow-up by body weight status at baseline.

|  | **Underweight/Normal Weight ^§^  # Follow up Visits = 3526** | | **Overweight and Not Obese ^§^  # Follow up Visits = 4028** | | **Obese ^§^  # Follow up Visits = 2829** | |
| --- | --- | --- | --- | --- | --- | --- |
|  | **OR (95% CI)** | ***p*** | **OR (95% CI)** | ***p*** | **OR (95% CI)** | ***p*** |
| **Serum 25(OHD) at baseline, nmol/L** |  |  |  |  |  |  |
| <50 | ref |  | ref |  | ref |  |
| 50–<75 | 0.82 (0.42, 1.58) | 0.55 | 0.73 (0.44, 1.19) | 0.20 | 0.76 (0.47, 1.23) | 0.27 |
| 75–<100 | 0.80 (0.43, 1.53) | 0.51 | 0.69 (0.40, 1.16) | 0.16 | 0.55 (0.32, 0.97) | **0.04** |
| 100–<125 | 0.64 (0.32, 1.30) | 0.22 | 0.53 (0.29, 0.98) | **0.04** | 0.24 (0.12, 0.51) | **<0.01** |
| ≥125 | 0.99 (0.47, 2.06) | 0.97 | 0.64 (0.33, 1.23) | 0.18 | 0.46 (0.20, 1.05) | 0.06 |
| **Change in serum 25(OH)D, nmol/L** |  |  |  |  |  |  |
| No improvement | ref |  | ref |  | ref |  |
| Increase of <25 | 1.25 (0.74, 2.10) | 0.40 | 0.87 (0.54, 1.39) | 0.56 | 0.91 (0.55, 1.48) | 0.69 |
| Increase of 25–<50 | 1.09 (0.62, 1.92) | 0.76 | 0.72 (0.44, 1.17) | 0.18 | 0.44 (0.26, 0.75) | **<0.01** |
| Increase of 50–<75 | 0.80 (0.43, 1.47) | 0.47 | 0.82 (0.48, 1.39) | 0.47 | 0.52 (0.29, 0.92) | **0.03** |
| Increase of ≥75 | 1.31 (0.73, 2.34) | 0.36 | 0.91 (0.54, 1.54) | 0.74 | 0.64 (0.36, 1.13) | 0.12 |

Abbreviations: OR, Odds Ratio; 95% CI, 95% Confidence Interval; CVD, cardiovascular disease; CRP, C-reactive protein; LDL-cholesterol, low density lipoprotein cholesterol; **^§^** Adjusted for elevated CRP at baseline, LDL-cholesterol at baseline, age at baseline, gender, blood pressure status baseline, smoking status at baseline, alcohol consumption status at baseline, and physical activity level at baseline, and change in physical activity level.
